# Verification in the Early Stages of the COVID-19 Pandemic: Sentiment Analysis of Japanese Twitter Users

**DOI:** 10.2196/37881

**Published:** 2024-02-06

**Authors:** Ryuichiro Ueda, Feng Han, Hongjian Zhang, Tomohiro Aoki, Katsuhiko Ogasawara

**Affiliations:** 1 Faculty of Health Sciences Hokkaido University Sapporo Japan; 2 Graduate School of Medicine Hokkaido University Sapporo Japan

**Keywords:** COVID-19, sentiment analysis, Twitter, infodemiology, NLP, Natural Language Processing

## Abstract

**Background:**

The COVID-19 pandemic prompted global behavioral restrictions, impacting public mental health. Sentiment analysis, a tool for assessing individual and public emotions from text data, gained importance amid the pandemic. This study focuses on Japan’s early public health interventions during COVID-19, utilizing sentiment analysis in infodemiology to gauge public sentiment on social media regarding these interventions.

**Objective:**

This study aims to investigate shifts in public emotions and sentiments before and after the first state of emergency was declared in Japan. By analyzing both user-generated tweets and retweets, we aim to discern patterns in emotional responses during this critical period.

**Methods:**

We conducted a day-by-day analysis of Twitter (now known as X) data using 4,894,009 tweets containing the keywords “corona,” “COVID-19,” and “new pneumonia” from March 23 to April 21, 2020, approximately 2 weeks before and after the first declaration of a state of emergency in Japan. We also processed tweet data into vectors for each word, employing the Fuzzy-C-Means (FCM) method, a type of cluster analysis, for the words in the sentiment dictionary. We set up 7 sentiment clusters (negative: anger, sadness, surprise, disgust; neutral: anxiety; positive: trust and joy) and conducted sentiment analysis of the tweet groups and retweet groups.

**Results:**

The analysis revealed a mix of positive and negative sentiments, with “joy” significantly increasing in the retweet group after the state of emergency declaration. Negative emotions, such as “worry” and “disgust,” were prevalent in both tweet and retweet groups. Furthermore, the retweet group had a tendency to share more negative content compared to the tweet group.

**Conclusions:**

This study conducted sentiment analysis of Japanese tweets and retweets to explore public sentiments during the early stages of COVID-19 in Japan, spanning 2 weeks before and after the first state of emergency declaration. The analysis revealed a mix of positive (joy) and negative (anxiety, disgust) emotions. Notably, joy increased in the retweet group after the emergency declaration, but this group also tended to share more negative content than the tweet group. This study suggests that the state of emergency heightened positive sentiments due to expectations for infection prevention measures, yet negative information also gained traction. The findings propose the potential for further exploration through network analysis.

## Introduction

### Background

The COVID-19 outbreak that occurred in December 2019 in Wuhan City, Hubei Province, China, spread rapidly in other countries after January 2020. Lockdowns were implemented primarily in Europe after March 2020 as infection prevention measures. The use of lockdowns as a quarantine measure varied from country to country; however, in the United States, the United Kingdom, France, and other countries, strict measures to regulate behavior were implemented, such as curfews and total school closures, with penalties imposed for violations.

COVID-19 spread rapidly in Japan after the first infection was confirmed on January 16, 2020, with incidents such as the mass infection on the Diamond Princess cruise ship in early February [1]. On April 7, the Japanese government declared a state of emergency in 7 prefectures—Tokyo, Kanagawa, Saitama, Chiba, Osaka, Hyogo, and Fukuoka—owing to the rapid spread of the infection by mass infection in medical facilities and elsewhere [2]. Although the restrictions imposed by the emergency declaration (eg, requests to remain inside and limitations on large-scale events) were less enforceable than those imposed by the lockdown, they did result in a significant decrease in travel rates throughout Japan. However, previous studies have shown that such strong behavioral restrictions may have a negative psychological impact on the public [3]. The emergency declaration was extended to all prefectures, and the restrictions imposed by the emergency declaration were subsequently lifted on May 25. Table 1 summarizes the major developments in the early stages of the COVID-19 outbreak in Japan in chronological order.

**Table 1:** Japan’s response to the initial spread of COVID-19.

Date	Events	References
2020/1/16	The first case of COVID-19 infection is confirmed in Kanagawa Prefecture, Japan.	[4]
2020/2/4	COVID-19 infection is confirmed in passengers on the Diamond Princess, a large cruise ship, returning to Hong Kong.	[1]
2020/2/27	The Japanese government requests the temporary closure of all elementary schools, junior high schools, and high schools in Japan from March 2 to spring break.	[5]
2020/3/10	The Japanese government declares the new coronavirus infection a historical emergency.	[6]
2020/3/13	The prime minister can now declare a “state of emergency.”	[7]
2020/3/26	The prime minister also orders the establishment of a government task force based on the act on special measures.	[8]
2020/4/7	The Japanese government declares a state of emergency. Seven prefectures (Tokyo, Kanagawa, Saitama, Chiba, Osaka, Hyogo, and Fukuoka), including the Tokyo metropolitan area, are designated as target areas.	[9]
2020/4/16	An emergency declaration is extended to cover all prefectures until May 6.	[10]
2020/5/4	A decision is made to extend the period of the state of emergency until May 31.	[9]
2020/5/14	The Japanese government decides to lift the state of emergency for 39 prefectures, excluding 8 prefectures on special alert (Tokyo, Kanagawa, Saitama, Chiba, Hokkaido, Kyoto, Osaka, and Hyogo).	[9]
2020/5/21	The Japanese government decides to the lift state of emergency for Kyoto, Osaka, and Hyogo.	[9]
2020/5/25	The Japanese government decides to lift the state of emergency for all prefectures.	[9]

### Prior Work in Infodemiology

Following the spread of COVID-19, social networking services (SNSs) were used to transmit information about the virus, accelerating activity in the field of infodemiology, which utilizes this data. Infodemiology is a relatively new research field that combines health informatics and public health with data analysis. It is a scientific discipline that studies the distribution of information and its determinants in information media, particularly the internet, to provide reliable information on public health [11]. Infodemiology became widely known after the World Health Organization (WHO) used the term at the first WHO Infodemiology Conference in response to the spread of COVID-19 and stated the need to promote research activities in this field worldwide [12]. In a previous study, Su et al [13] used sentimental analysis of text information from SNS data to reflect public concerns and psychological changes in individuals, providing information to promote public health. In particular, a sentiment analysis of the Italian region of Lombardy, where the lockdown was enforced, indicated that the number of SNS users with feelings of “anxiety” decreased after the lockdown. In addition, Heras-Pedrosa et al [14] observed through sentiment analysis that “anxiety” and “anger” toward government policies were the top feelings in Spain in the early stages of the infection. Furthermore, in Japan, Niu et al [15] conducted a sentiment analysis from SNS text data on the reasons for the delay in COVID-19 vaccine uptake compared to other countries, suggesting that concerns about side effects may have outweighed the fear of infection in the initial vaccination process. Thus, social media–based analysis reflects the psychological changes in individuals and enables the provision of real-time information to the government enacting public health policies and infection prevention measures.

### SNS Usage in Japan

The importance of social media has been increasing in Japan as well, with social media being utilized in public health countermeasures against recent pandemics. The usage rate of SNSs in Japan is still on the rise, with the Ministry of Internal Affairs and Communications’ 2020 Survey on Communications Usage Trends [16] showing that the percentage of people using SNSs was 73.8%, an increase of 4.8% from the previous year. It also points out that the growth is particularly large in the age groups comprising people 19 years and below and 60 years and above, indicating that the usage rate of SNSs by age group is increasing for all generations. In terms of the purpose of use, the second-highest percentage of respondents chose “to search for information I want to know,” followed by “to communicate with acquaintances,” suggesting that social media is used by all generations in Japan as an important means of obtaining information. However, while the research field of infodemiology is being actively promoted, there are limited reports on infodemiology in Japan, even though social media is used by a wide range of generations.

### Study Purpose

In this study, we investigated psychological changes in individuals after the initial spread of COVID-19 in Japan and public sentiment changes following state-of-emergency declarations by conducting sentiment analysis using SNS data in infodemiology.

## Methods

### Research Data

We extracted geocoded Twitter data using “Nazuki no Oto,” a service provided by NTT Data Corporation [17]. The target period was from midnight on March 23, 2020, 2 weeks before the first declaration of a state of emergency in Japan, to April 21, 2020. We selected tweets containing the keywords “コロナ(corona),” “COVID-19,” and “新型肺炎 (new pneumonia)” by random sampling of 4,997,353 tweets. In addition, the data used in this study include retweets, a function that allows users to repost other users’ or their own tweets. Duplicate tweets were removed from the Twitter data extracted for this study, and only unique Twitter data were used.

### Data Preprocessing

Before conducting the sentiment analysis on the extracted Twitter data, we preprocessed the data. For preprocessing, we deleted Twitter data that contained symbols that could not be analyzed by morphological analysis, hashtags (eg, #COVID-19), and URLs only. Consequently, a total of 4,965,100 tweets were used as the target data for sentiment analysis.

### Morphological Analysis

In contrast to structured and quantitative data, which can be easily analyzed by a computer, qualitative text data, which are often used in sentiment analysis, require processing to extract the data objectively. Therefore, unstructured data are analyzed to convert them from qualitative to quantitative data. However, thus far, analyzing qualitative data in Japanese has been considered a difficult task. One reason for this is that Japanese grammar is more complex than English and other languages [18]. However, with the recent development of natural language processing, it is possible to separate sentences naturally and convert them into quantitative data on a practical level by preparing Japanese dictionary functions for Japanese text data. Morphological analysis determines the smallest grammatically meaningful unit that constitutes a sentence by demarcating the boundaries of words and phrases in the text data. Following decomposition, the part of speech and the type of conjugation are determined by referring to a registered dictionary. In this study, we used a morphological analyzer, MeCab (version 0.996; Kyoto University).

The International Phonetic Alphabet (IPA) dictionary, integrated within the Japanese morphological analysis system Chasen, is widely used for performing morphological analysis in MeCab [19]. However, conventional IPA dictionaries are limited in their ability to support conventional Japanese words and phrases and do not support neologisms and phrases unique to Japanese. To solve this problem, a new system dictionary called mecab-ipadic-NEologd was introduced [20]. This dictionary is updated every Monday and Sunday and can be automatically updated and registered from websites, such as news sites and social media. Therefore, the dictionary can handle text data on the web where unique expressions and new words are frequently used. In this study, we registered mecab-ipadic-NEologd and performed morphological analysis on text data from the SNS Twitter because many unique expressions and new words are used there.

### Japanese Sentiment Dictionary

We utilized the Japanese Linguistic Inquiry and Word Count (JIWC) dictionary (Nara Institute of Science and Technology) for the sentiment analysis, employing cloud sourcing to access the latest corpus. This Japanese emotional dictionary was used for determining emotions in sentiment analysis, encompassing 7 categories: “anger,” “concern,” “disgust,” “sadness,” “surprise,” “trust,” and “joy” [21]. Examples of words in the Japanese emotion expression dictionary are shown in Table 2. Among the emotions, “trust” and “joy” were selected as positive emotions, and “anger,” “anxiety,” “disgust,” and “sadness” were selected as negative emotions based on previous studies [22].

**Table 2:** Examples of words included in the JIWC^a^ dictionary.

Sentiment	Examples of words
Anger	怒った (angry), 怒り(rage), 悪い(bad), 嫌がらせ(harassment), イライラ(irritation), うるさい (noisy), ゴミ (garbage), 暴言(rant), 煽り (aggravation), 理不尽な (unreasonable), 騒音 (noise), 迷惑 (annoyance), 被害 (damage), 虐待(abuse), 裏切り (betrayal)
Anxiety	不安(anxious), 不安だ (worrying), 不安な(anxiety), 病 (illness), 症状(symptom), このまま (at this rate), この先 (from now on ), 考える (thinking)
Disgust	嫌いな (dislike), 嫌がらせ (harass), 嫌な (disgust), うるさい (loud), テロ (terror), 犯罪 (crime), 犯人 (criminal), ひどい (terrible), 悪 (evil), 悪かった (bad), 批判 (criticize), 無い (no), 無し (none), 無視 (ignore), 嘘 (lie), 汚い (dirty)
Sad	悲しい (sad), 悲観 (pessimistic), 悲愁 (melancholy), 哀感 (sorrowful), 哀傷 (piteous), 泣き (weeping), 泣き叫ぶ (wailing), 嘆き (lamenting), 涙 (tears), 涙声 (tearful), 追悼 (mourning), 痛嘆 (painful), センチメンタル (sentimental)
Surprise	いきなり (suddenly), サプライズ (surprise), びっくり (surprised), 偶然 (accidentally), 知った (learned), 知って (knew), 解散 (dissolved), 詐欺 (fraud), 発見 (discovered)
Trust	仲間 (companion), 任せ (entrust), 依頼 (request), 信用 (trust), 頼り (rely), 頼んで (ask), 助け (help), 守って (protect), 親友 (friend), 親身に (friendly), 関係 (relationship), サポート (support), フォロー (follow)
Joy	遊び (play), 遊んで (playing), 楽しい (fun), 出かけた (went out), おいしい (delicious), 食事 (meal), できた (could), 会って (meet), 会話 (conversation), 笑い (laugh), 笑顔 (smile), 好きな (like)

^a^JIWC: Japanese Linguistic Inquiry and Word Count.

### Data Clustering

The sentiment analysis conducted in this study involved determining emotions in Twitter data by comparing the words in the text with those found in the JIWC dictionary. However, since the words after the morphological analysis were unstructured data, it was not possible to perform numerical calculations to assess their similarity to the words in the dictionary. To address this issue, we used Word2Vec processing to vectorize the text data for both Twitter data and the Japanese emotional dictionary.

Word2Vec is a model proposed by Mikolov et al [23,24] that represents word meanings using low-dimensional vectors, enabling semantic calculations in natural language processing. When vectorizing a large amount of text data, as in this study, individually vectorizing each word can result in an enormous number of dimensions, making it impractical in terms of computation time. Therefore, Word2Vec enables the vectorization of large text data through an inference-based approach using neural networks. Inference-based methods involve making predictions about what goes into a word when given its context (the surrounding words in a sentence). For example, when given the sentence “You ??? goodbye, and I say hello,” we can easily infer that the missing word is “say.” In this case, the context for “???” consists of 2 words: “you” and “goodbye.” The challenge is to infer what fits into that word based on the surrounding context, and thus learn word occurrence patterns. This approach is based on the distributional hypothesis, which suggests that word meanings are formed by the context of the surrounding words rather than inherent in the words themselves. Word2Vec includes 2 models, namely, the continuous bag-of-words (CBOW) model and the skip-gram model, to solve this inference issue. Generally, the skip-gram model is considered to have higher model accuracy after training, but it incurs higher computational costs since it needs to calculate losses for each context. This study’s text data comprises millions of individual pieces, and due to the added morphological analysis, a higher number of words per sentence was anticipated. Therefore, we anticipated that the computational cost for predictions would become immense. As a result, we employed the CBOW model for word embedding processing. After the data collected from Twitter and the terms registered from each Japanese sentiment dictionary were vectorized, Fuzzy-C-Means (FCM) was used to cluster each of the 7 sentiments.

The FCM method is a nonhierarchical soft clustering technique based on fuzzy logic theory. Fuzzy logic theory, originating from the concept of fuzzy sets proposed by LA Zadeh in 1965, provides a framework for quantitatively handling uncertainty and ambiguity in human subjective thinking and decision-making. FCM is a soft clustering method that applies fuzzy logic theory to cluster data [25]. In traditional hard clustering, data are assigned to clusters by being represented as either belonging (1) or not belonging (0) to a specific cluster. In contrast, because FCM is a soft clustering method, it allows data to partially belong to multiple clusters, such as 0.8 belonging to one cluster and 0.2 belonging to another. FCM clustering is carried out using the following algorithm. The membership values, representing the degree to which data points belong to different clusters, are considered:



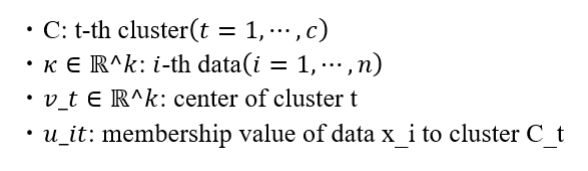



In this case, the following conditions are satisfied:



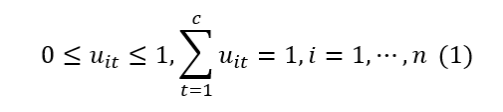



The matrix *U*, denoted as [*u_it*], represents an n×c matrix with the membership value *u_it* as an element. Meanwhile, the matrix *V*, represented as [*v_t*], is an n×c matrix with cluster center *v_t* as an element.

Bezdek proposed the following formula for the FCM model that minimizes the objective function by the weighted sum of the Euclidean squared distances between each data and the center of each cluster under the condition of (1) [26]:



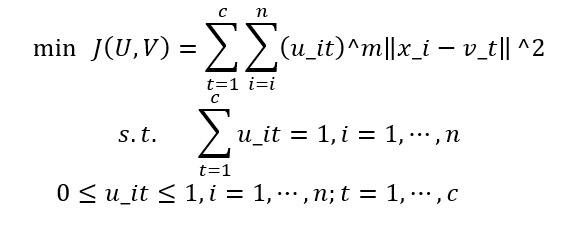



Here, *m* is a fuzzy coefficient parameter (*m*>1) that adjusts the strength of ambiguity. When *m*=1, the FCM model corresponds to the hard clustering k-means model. In this case, the objective function *J(U, V)* is linearized with respect to *u_it*, eliminating soft clustering. FCM clustering is carried out through the following steps. First, given a data set {x_1, ⋯, x_n}, we determine the number of clusters *t* (2 ≤ t ≤ c) and the parameter m ∈ (1, ∞). Next, we initialize the membership values *u_it* with U^(0) = {u_it^0} randomly. We provide a sufficiently small positive number ε to determine the termination of the loop. Second, we use the current membership values u_it to calculate the cluster centers v_t^p using the following formula:



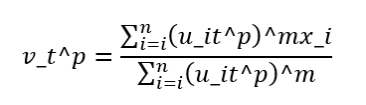



Third, we update the membership values from *u_it*^*p* to *u_it*^(*p*+1) using the following formula:



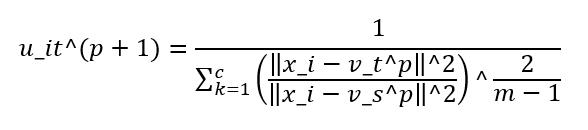



Finally, if *‖u_it*^(*p*+1) - *u_it*^*p‖* < ε holds for all *i* and *t*, we terminate the loop. Otherwise, we increment *p* by 1 and return to the second step. Once the loop terminates, we obtain the center points for each cluster and the membership values for each sample data, completing the clustering process. In this study, FCM was used on text data to reduce the number of words included in an emotion dictionary and construct the emotion dictionary, allowing for more accurate sentiment analysis of the text data due to the influence of a single word on multiple emotions. Both tweets and retweets of Twitter data were used, and quoted retweets, which are retweets of others’ posts with additional comments, were also included.

**Figure 1:**
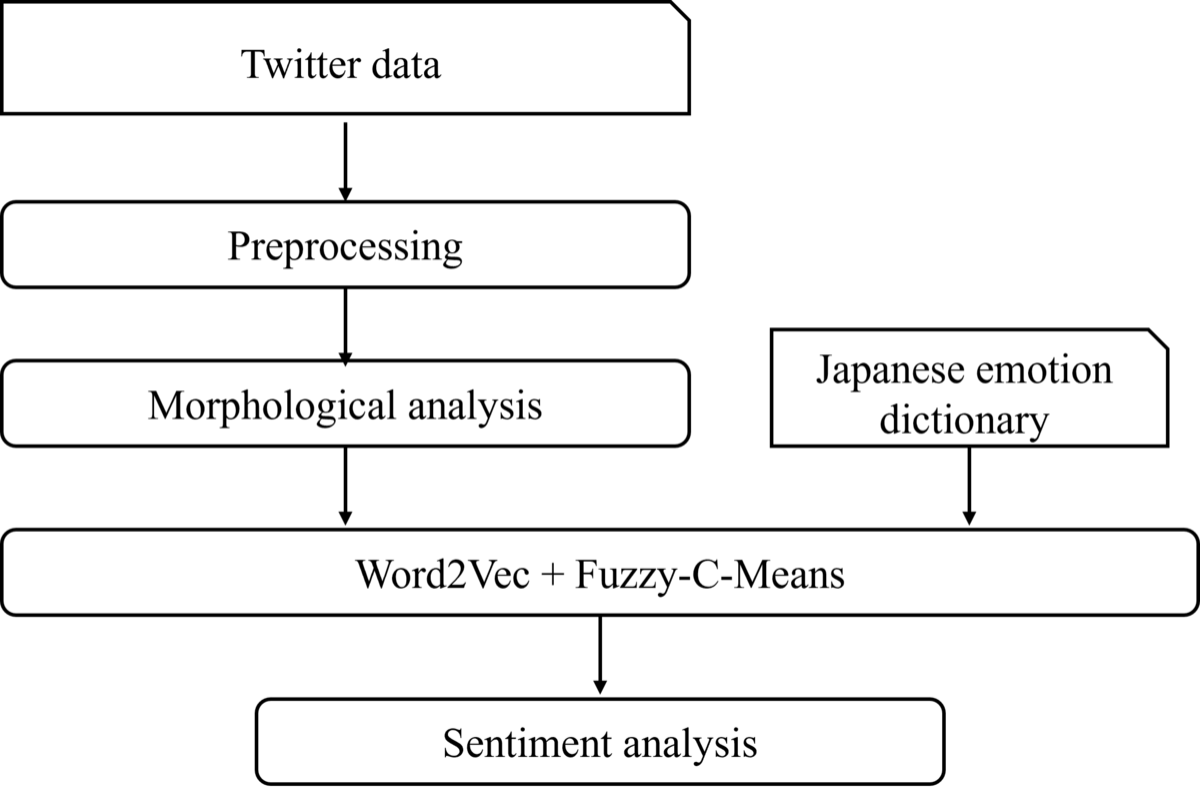
Sentiment analysis flowchart.

**Figure 2:**
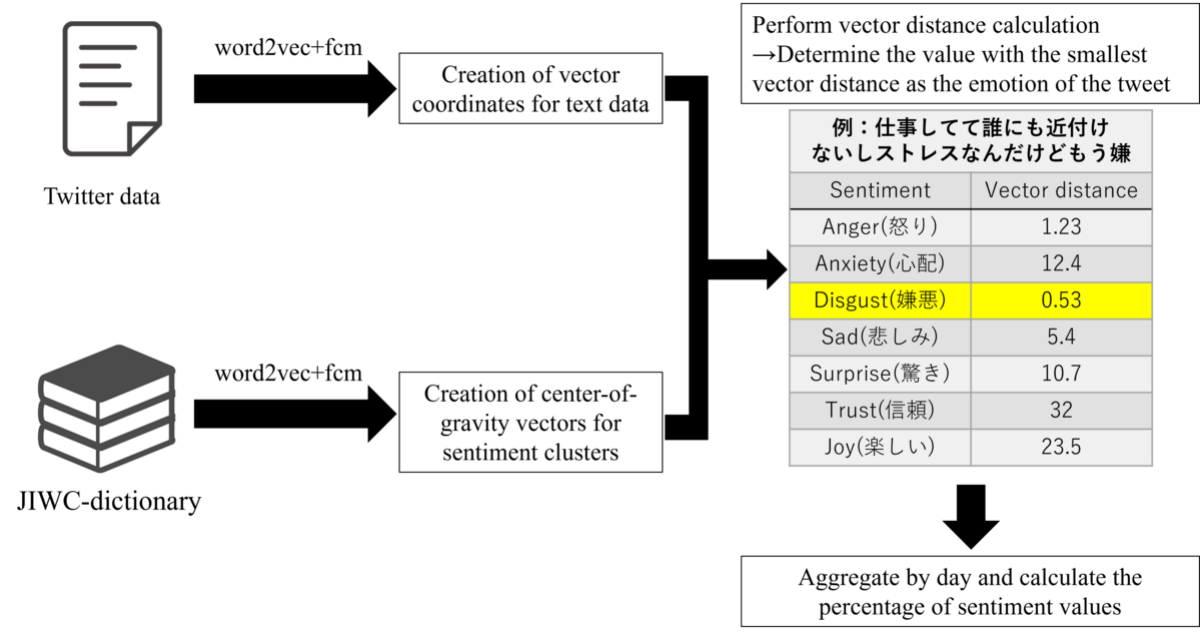
Diagram of the sentiment determination method. FCM: Fuzzy-C-Means; JIWC: Japanese Linguistic Inquiry and Word Count.

After vectorization using Word2Vec and clustering using the FCM method, the distance between the vector coordinates of each tweet and the center-of-gravity vector of each written sentiment was calculated. Next, the value with the shortest vector distance was determined as the sentiment of that tweet. The entire sentiment analysis in this study was performed using the Python programming language (version 3.9.4). A path diagram of the overall sentiment analysis is shown in Figure 1, and a summary diagram of the sentiment determination method is shown in Figure 2.

### Examining Sentiment Changes Before and After the State of Emergency Declaration

The Twitter data were categorized into 2 groups: the tweet group and the retweet group. The study period was divided into “before the declaration of a state of emergency,” which ranged from midnight on March 23, 2020, until PM 11:59:59 on April 6, 2020, and “after the declaration of a state of emergency,” which ranged from midnight on April 7, 2020, to PM 11:59:59 until April 21, 2020. We calculated the proportion of emotions before and after the declaration of a state of emergency in both the tweet and retweet groups. The sentiment analysis results were validated using 2 methods. The first method involved comparing emotions using a between-group comparison of 7 emotions over approximately 2 weeks before and after the declaration of a state of emergency. This comparison was based on daily average values for each emotion. The second method involved dividing the data into two groups: (1) the tweet group, consisting of posts made by the users themselves, and (2) the retweet group, consisting of posts shared for the purpose of dissemination. Sentiment analysis results were aggregated daily, classifying the data as either positive (“trust” and “joy”) or negative (“anger,” “concern,” “disgust,” and “sadness”) and then comparing the tweet and retweet groups. Both methods conducted a median difference examination using the Mann-Whitney U test, with statistical significance set at *P*<.05, utilizing the statistical software JMP (version 16.0; SAS).

### Ethical Considerations

This study was conducted while adhering to strict ethical considerations and did not require ethics approval. To avoid identification of personal information, the Twitter data used were limited to the type of post (tweet or retweet), text, and the date and time of the post for data analysis. The data used did not contain any personally identifiable information. In addition, efforts were made to ensure transparency throughout the design and conduct of this study.

## Results

### Research Data

We were able to judge sentiment through the sentiment analysis in 4,884,297 (97.74%) cases out of a total of 4,997,353 cases. In addition, the number of tweets was 1,374,025 (28.13%), and the number of retweets was 3,510,272 (71.87%). The number of tweets and retweets per day is shown in Table 3, and the daily trends for the data from March 23, 2020, to April 21, 2020, are shown in Multimedia Appendix 1.

**Table 3:** Daily tweet and retweet counts.

Date	Tweets (n=1,374,025), n (%)	Retweets (n=3,510,272), n (%)
2020/3/23	4666 (0.34)	13,643 (0.39)
2020/3/24	25,067 (1.82)	71,040 (2.02)
2020/3/25	33,759 (2.46)	87,476 (2.49)
2020/3/26	41,944 (3.05)	115,842 (3.30)
2020/3/27	39,433 (2.87)	103,798 (2.96)
2020/3/28	37,160 (2.70)	106,915 (3.05)
2020/3/29	37,804 (2.75)	108,868 (3.10)
2020/3/30	74,353 (5.41)	209,297 (5.96)
2020/3/31	51,765 (3.77)	144,594 (4.12)
2020/4/1	48,902 (3.56)	121,864 (3.47)
2020/4/2	48,127 (3.50)	119,259 (3.40)
2020/4/3	52,918 (3.85)	123,835 (3.53)
2020/4/4	48,470 (3.53)	113,346 (3.23)
2020/4/5	54,358 (3.96)	115,172 (3.28)
2020/4/6	75,831 (5.52)	175,918 (5.01)
2020/4/7	76,184 (5.54)	195,158 (5.56)
2020/4/8	60,645 (4.41)	179,707 (5.12)
2020/4/9	55,231 (4.02)	156,760 (4.47)
2020/4/10	51,078 (3.72)	134,393 (3.83)
2020/4/11	44,901 (3.27)	111,213 (3.17)
2020/4/12	42,403 (3.09)	96,575 (2.75)
2020/4/13	42,117 (3.07)	107,539 (3.06)
2020/4/14	42,800 (3.11)	105,344 (3)
2020/4/15	44,185 (3.22)	118,456 (3.37)
2020/4/16	48,618 (3.54)	122,458 (3.49)
2020/4/17	44,494 (3.24)	132,009 (3.76)
2020/4/18	38,270 (2.79)	111,351 (3.17)
2020/4/19	38,872 (2.83)	110,308 (3.14)
2020/4/20	39,611 (2.88)	116,187 (3.31)
2020/4/21	30,059 (2.19)	78,522 (2.24)

### Percentage of Emotions in the Sentiment Analysis

**Figure 3:**
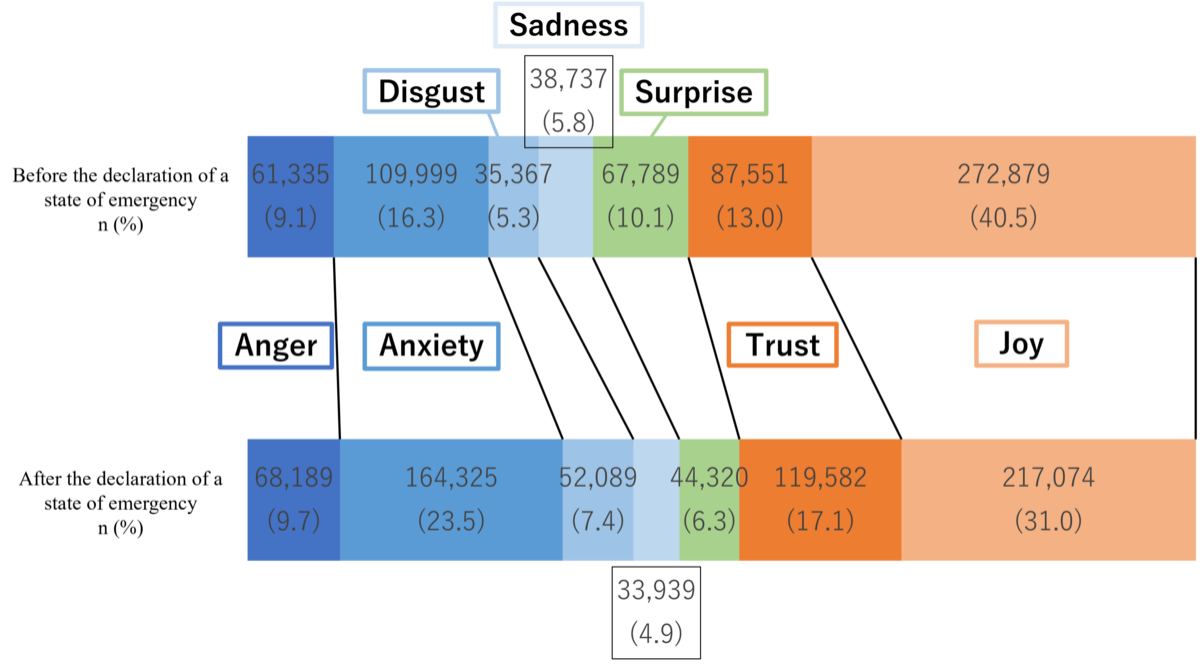
Sentiment analysis value ratio in the tweet group.

**Figure 4:**
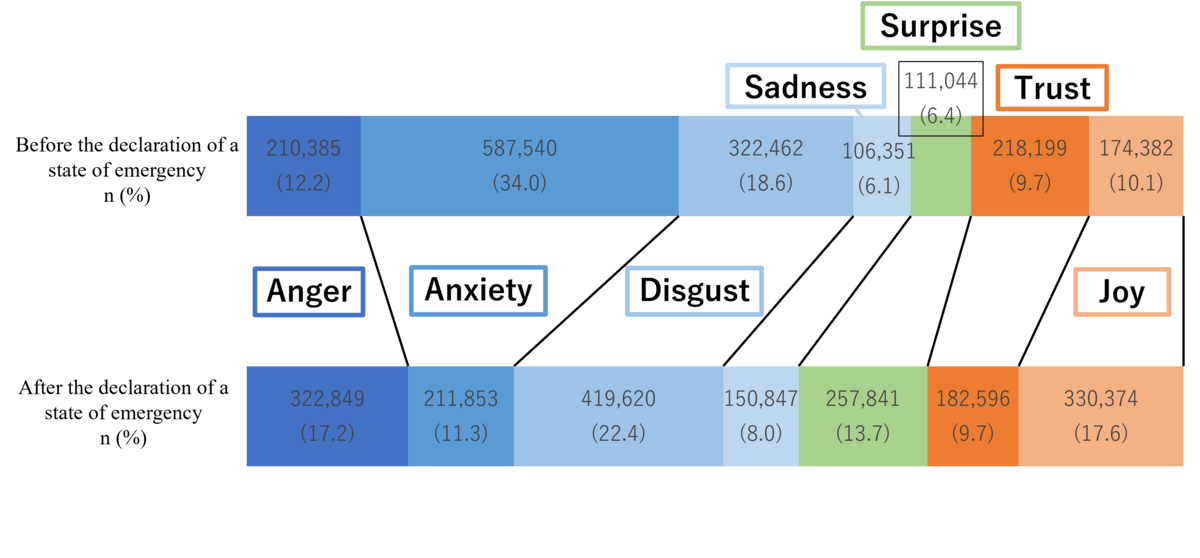
Sentiment analysis value ratio in the retweet group.

The results of the sentiment analysis on the tweet and retweet groups for the period between midnight on March 23, 2020, to 23:59:59 on April 6, 2020 (before the declaration of the state of emergency) are shown in Figure 3. The results for the period between midnight on April 7, 2020, and 23:59:59 on April 21, 2020 (after the declaration of the state of emergency) are shown in Figure 4. In the tweet group, the positive emotion “joy” was highest both before and after the state of emergency declaration at 40.5% (n=272,879) and 31% (n=217,074), respectively, while in the retweet group, the negative sentiment of “worry” was 34% (n=587,540), and “disgust” was 18.6% (n=322,462) during the period before the state of emergency declaration. These percentages were higher than those for the other emotions.

### Changes in Sentiment Before and After the Declaration of a State of Emergency

Table 4 shows the results of the sentiment analysis yielding the proportions of the 7 emotion types before and after the declaration of the state of emergency. The Mann-Whitney U test comparison of differences in median values revealed that the sentiment of joy significantly increased in the retweet group (*P*<0.05). However, no significant differences were observed for the other emotions.

**Figure 5:**
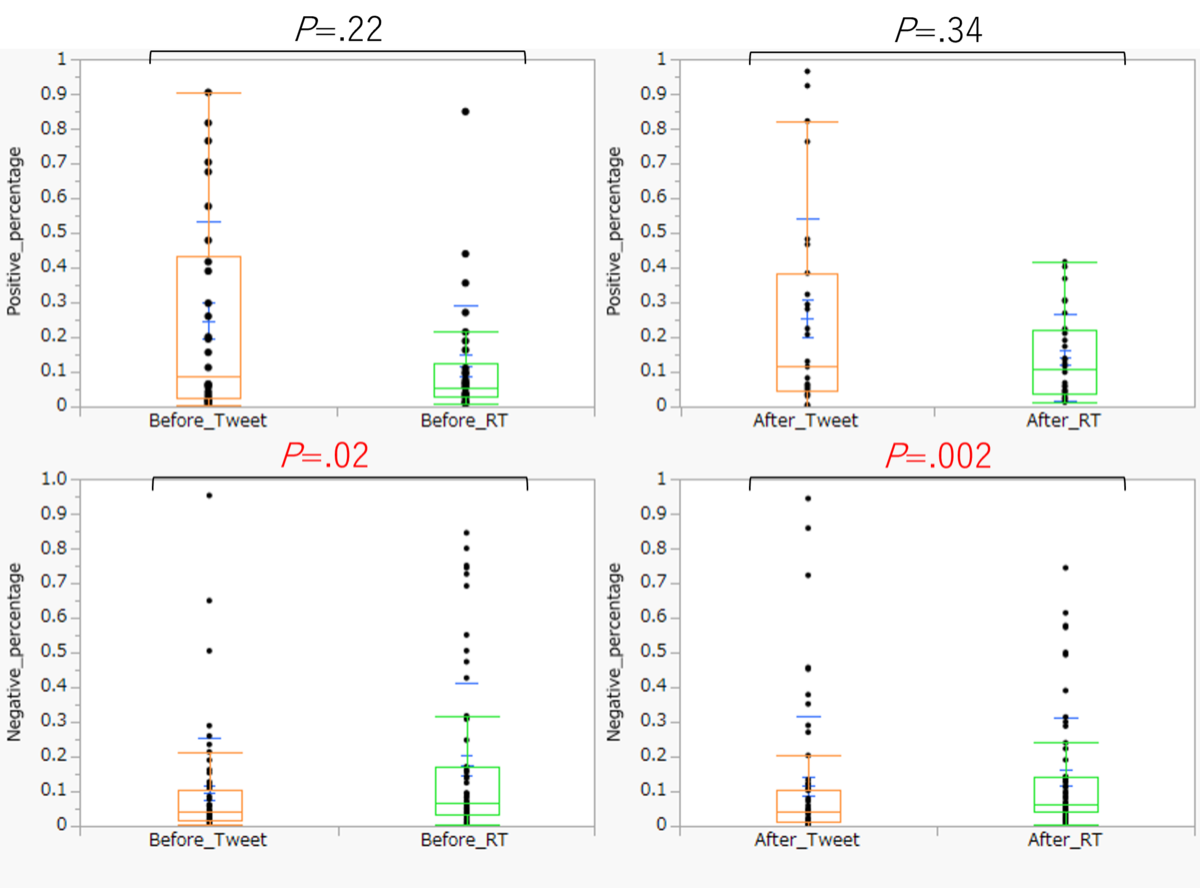
Graphs displaying the results from a comparative study illustrating changes in positive and negative sentiments between the tweet and retweet groups. RT: retweet.

Table 5 and Figure 5 show the results of testing the change of positive and negative content between the tweet group and retweet groups. In the 2 weeks before and after the emergency declaration, the retweet group tended to post more negative content than the tweet group (before *r*=0.29, *P*=.02; after *r*=.0.40, *P*=.002). However, there was no difference between the tweet and retweet groups in the percentage of positive responses.

**Table 4:** Sentiment changes before and after the state of emergency declaration^a^.

Sentiments	Before (n=15)			After (n=15)			*P* value
	Median	SD	Median		SD		
Anger tweet	0.042	0.061	0.024		0.082	.80	
Anger retweet	0.042	0.051	0.063		0.035	.84	
Anxiety tweet	0.063	0.078	0.050		0.135	.90	
Anxiety retweet	0.210	0.307	0.054		0.052	.25	
Disgust tweet	0.023	0.293	0.021		0.020	.59	
Disgust retweet	0.073	0.136	0.127		0.100	.43	
Sadness tweet	0.041	0.025	0.035		0.025	.51	
Sadness retweet	0.041	0.045	0.055		0.025	.28	
Surprise tweet	0.038	0.090	0.016		0.022	.16	
Surprise retweet	0.051	0.023	0.035		0.104	.93	
Trust tweet	0.035	0.033	0.038		0.056	.32	
Trust retweet	0.059	0.032	0.061		0.050	.80	
Joy tweet	0.390	0.258	0.281		0.191	.62	
Joy retweet	0.041	0.057	0.191		0.097	.04	

^a^Before refers to the period from midnight on March 23, 2020, until 11:59:59 PM on April 6, 2020, while after refers to the period from midnight on April 7, 2020, until 11:59:59 PM on April 21, 2020.

**Table 5:** Comparison results of positive and negative changes between the tweet and retweet groups.

Sentiment		Tweet		Retweet		*P* value
		Median	SD	Median	SD	
**Positive (n=30)**						
	Before	0.089	0.286	0.054	0.174	.22
	After	0.099	0.290	0.108	0.123	.34
**Negative (n=60)**						
	Before	0.040	0.161	0.066	0.236	.02
	After	0.038	0.202	0.063	0.173	.002

## Discussion

### Principal Findings

The Japanese language sentiment analysis conducted during this study’s target period, both before and after the declaration of the state of emergency, revealed that “joy,” associated with a “positive” sentiment, accounted for high proportions within the tweet group at 40.5% (n=272,879) before and 31% (n=217,074) after. On the other hand, “anxiety” and “disgust,” which express “negative” feelings, accounted for high percentages in both the tweet and retweet groups, especially in the retweet group, where “anxiety” accounted for 34% (n=587,540) and “disgust” accounted for 18.6% (n=322,464) of the total retweets before the state of emergency was declared. The self-restraint approach regulating behavior during the declaration of a state of emergency in Japan allowed movement across prefectures. This may have been a contributing factor to the widespread negative posts related to movements from the target area. This surge in negative sentiment was countered by a simultaneous rise in positive emotions, attributed to the anticipation of infection prevention following the state of emergency declaration. During the early stages of the COVID-19 spread in other countries, a previous study on English-speaking users indicated elevated levels of positive emotions linked to anticipations for potential policies [22]. A generally similar emotional response was apparent among the public in other countries. In the early stages of the spread of infection, when no vaccine or other countermeasures had been implemented, feelings of anxiety may have been expressed on social media, as well as expectations for strong countermeasures, such as behavioral restrictions. In contrast, the results of the sentiment analysis of English-language tweets corresponding to the same period showed that negative and positive emotions accounted for approximately the same proportions by late March, the end of the period covered in this study. Notably, the negative emotion “fear” occupied a higher percentage than other emotions around January and February [27]. In China and European countries, the first cases of infection were confirmed earlier than in Japan (where the initial expansion of the outbreak occurred in late March). Thus, the earlier spread of infection in those nations may have a significant impact on the sentiment analysis.

### Comparative Study Between the Tweet and Retweet Groups

When comparing the tweet and retweet groups, the retweet group tended to post more negative sentiments. In this regard, a previous study revealed that in the early stage of the COVID-19 outbreak among English-speaking users, many tweets had a positive sentiment, while many retweets had a negative sentiment [28]. It is clear that much of the information users wished to disseminate was negative in nature. As for the difference between groups in this study, there is a research report that focuses on virality, one of the characteristics of sentiment analysis using social media [29] Virality is an explosive spread of attention and information through social media and word-of-mouth on the internet. Virality is derived from “viral”—as in a virus. Previous research indicates that negative posts increase virality, while positive posts decrease virality. Therefore, for topics that attract substantial public attention, such as COVID-19, the topic of this study, there is a tendency to spread negative content in retweets, consequently increasing virality. This suggests a noteworthy contrast between the tweet and retweet groups.

### Limitations

There are a few key limitations of this study. First, the social media platform Twitter, which was used for the sentiment analysis in this study, had an age bias. According to a survey conducted by the Ministry of Internal Affairs and Communications in 2020, the Twitter usage rate is highest among teenagers (67.6%) and twentysomethings (79.8%) [30].

Additionally, data from the Ministry of Internal Affairs and Communications indicate that the usage rate declines with increasing age, especially among individuals aged 40 years and older. This suggests that the younger generation is the predominant user of Twitter as a whole. This suggests that the younger generation predominantly constitutes the main users of Twitter overall. Therefore, the results of the sentiment analysis in this study are not necessarily representative of the entire nation. In addition, the Twitter data used in this study were limited to Japanese-language content. We did not use location-based information or conduct analyses based on geographical data. As such, this data may originate from disproportionate samples depending on the prefecture. During Japan’s initial state of emergency declaration in 2020, the target areas comprised 7 prefectures: Tokyo, Kanagawa, Chiba, Saitama, Osaka, Kobe, and Fukuoka. Subsequently, on April 16, 2020, the target area was expanded to the entire country [9]. Throughout the study period covered, only some of the target areas were declared as emergency areas; therefore, emotional variations in Twitter usage may exist depending on the location of the users.

Second, the sentiment analysis categorized each tweet into one of 7 predefined sentiment types, limiting its ability to capture multiple sentiments, such as “anger” and “surprise,” within a single tweet or account for cases where the selected sentiments might not apply.

The Twitter data utilized in this study underwent random sampling for both tweets and retweets. Twitter incorporates a function known as “bot,” which automatically generates tweets in response to specified times and keywords. Numerous accounts, commonly referred to as “bot accounts,” are responsible for automatic posting. Shi et al [31] conducted a sentiment analysis on Twitter focusing on the #coronavirus hashtag from January 2020 to March 2020, including human and bot-generated tweets. Their findings revealed that bot-posted tweets had more negative sentiments compared to those posted by humans concerning the topic of COVID-19. This suggests that the bot feature intentionally promotes negative public opinion and sentiment. Consequently, it is plausible that the inclusion of a substantial amount of data posted by bot accounts in this study may have influenced the results of the sentiment analysis. Unfortunately, we were unable to preprocess the data to account for this aspect. For our future research, we anticipate that carrying out a network analysis using the results of this study will provide a deeper understanding of the specific subjects that capture public interest. In terms of social network analysis, Seungil [32] investigated how Twitter users in the United States accessed COVID-19–related information based on their posted data. The investigation revealed that during the initial outbreak period, users expressed significant concerns about the number of infections Additionally, the study highlighted that users were more likely to obtain COVID-19 information from news channel accounts and the official accounts of the president. Sakun et al [22] conducted a network analysis to uncover topics associated with different emotions based on the results of a sentiment analysis using Twitter text data They found that words like “pneumonia,” “influenza,” “infectious disease,” and “quarantine” were frequently linked to the emotion of “fear.” In addition, words like “pandemic,” “disease,” and “hospital” were associated with the emotion “sadness.” These results suggest that Twitter data can be used to understand the public’s awareness of and emotions toward pandemics, providing valuable insights for governmental responses. Hence, the results of the sentiment analysis should be used for further exploration in infodemiology, specifically by conducting a network analysis focusing on the topics associated with each sentiment identified in this study.

### Conclusions

In this study, we conducted a sentiment analysis using Japanese tweet and retweet text data spanning approximately 2 weeks before and after the first state of emergency declaration in Japan to assess public sentiments toward the initial spread of COVID-19. We observed a combination of positive sentiments (“joy”) and negative sentiments (“anxiety” and “disgust”) during the target period. The results of the Mann-Whitney U test indicated that feelings of joy significantly increased in the retweet group before and after the state of emergency declaration. However, there was a significant tendency for the retweet group to post more negative content compared to the tweet group. After the first state of emergency declaration, the anticipation regarding infection prevention measures due to this declaration contributed to an increase in positive sentiments. Moreover, it appears that information, including negative content, was more likely to be disseminated on the topic of COVID-19. Based on the results of this study, we believe that further development through network analysis is possible.

## Appendix

Multimedia Appendix 1 Total number of tweets and retweets per day.

## Abbreviations

CBOW: continuous bag-of-words
FCM: Fuzzy-C-Means
IPA: International Phonetic Alphabet
JIWC: Japanese Linguistic Inquiry and Word Count
SNS: social networking service
WHO: World Health Organization
