# Correction: Verification in the Early Stages of the COVID-19 Pandemic: Sentiment Analysis of Japanese Twitter Users

**DOI:** 10.2196/57880

**Published:** 2024-03-14

**Authors:** Ryuichiro Ueda, Feng Han, Hongjian Zhang, Tomohiro Aoki, Katsuhiko Ogasawara

**Affiliations:** 1 Faculty of Health Sciences Hokkaido University Sapporo Japan; 2 Graduate School of Medicine Hokkaido University Sapporo Japan

In “Verification in the Early Stages of the COVID-19 Pandemic: Sentiment Analysis of Japanese Twitter Users” (JMIR Infod 2024;3(1):e37881) the authors made 3 corrections.

1. The authorship list was previously listed as:

Ryuichiro Ueda, MA; Feng Han, MA; Hongjian Zhang, MD; Tomohiro Aoki, MA; Katsuhiko Ogasawara, Prof Dr

And has now been changed to:

Ryuichiro Ueda, MHA; Feng Han, MHA; Hongjian Zhang, PhD; Tomohiro Aoki, MHA; Katsuhiko Ogasawara, MBA, PhD

2. Author Feng Han’s affiliation was originally:

Faculty of Health Sciences, Hokkaido University, Sapporo, Japan

And was changed to:

Graduate School of Medicine, Hokkaido University, Sapporo, Japan

3. The phone number listed for the corresponding author was originally:

81 011 716 2111

And was changed to:

81 11 706 3409

The correction will appear in the online version of the paper on the JMIR Publications website on March 14, 2024 together with the publication of this correction notice. Because this was made after submission to PubMed, PubMed Central, and other full-text repositories, the corrected article has also been resubmitted to those repositories.

