# Supplementary figures and images for: Verification in the Early Stages of the COVID-19 Pandemic: Sentiment Analysis of Japanese Twitter Users
Source: JMIR Infodemiology. 2024 Feb 6;4:e37881. doi: 10.2196/37881 (PMC10849083; doi:10.2196/37881)

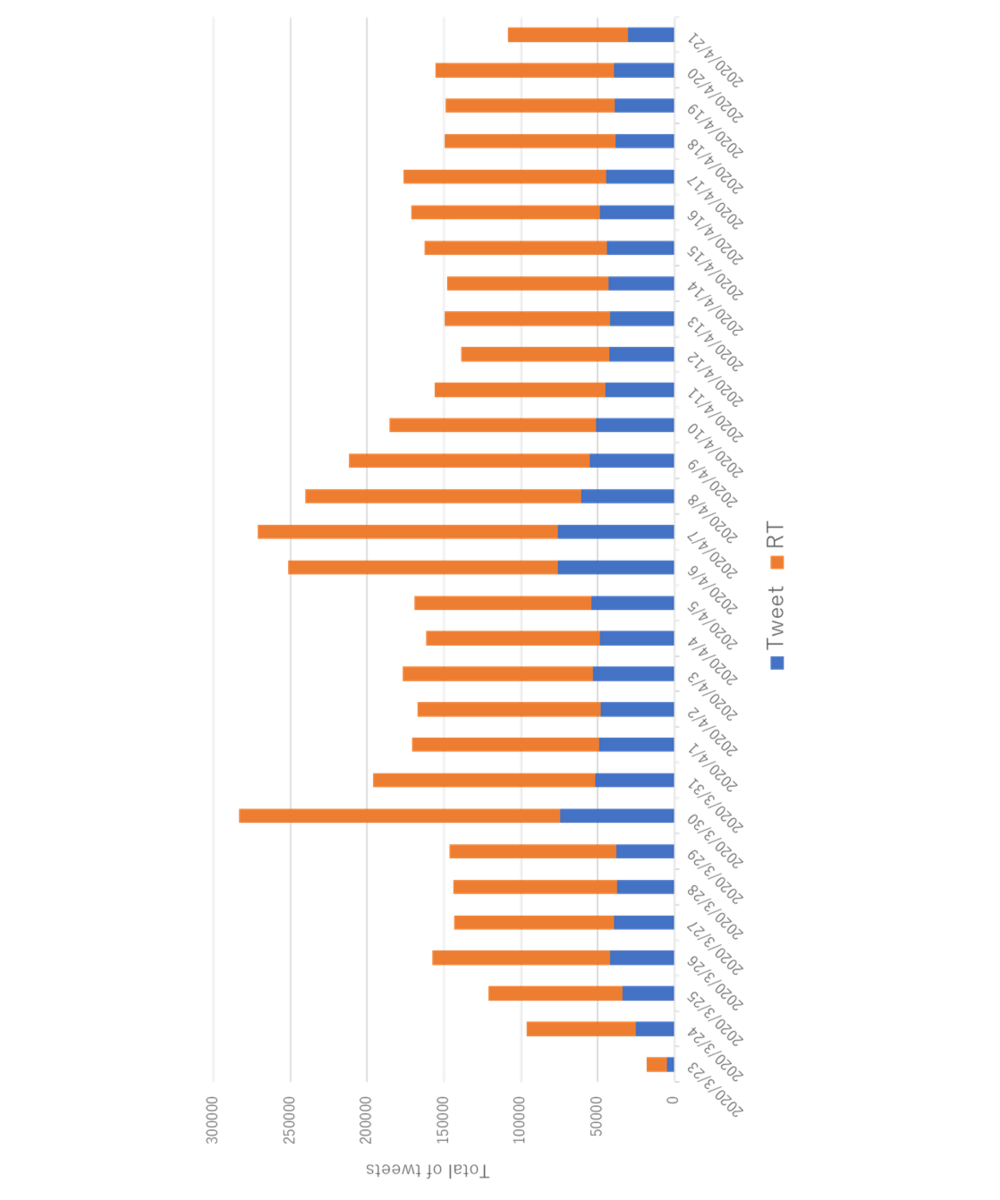

Supplement: Multimedia Appendix 1 [file infodemiology_v4i1e37881_app1.png]
